# Gluten‐free Nan‐e‐Fasaee: Formulation optimization on the basis of quinoa flour and inulin

**DOI:** 10.1002/fsn3.3829

**Published:** 2023-11-27

**Authors:** Narjes Jamali, Mehran Sayadi, Roghayeh Nejati, Faezeh Mohammadi, Amene Nematollahi, Neda Mollakhalili‐Meybodi

**Affiliations:** ^1^ Student Research Committee Fasa University of Medical Sciences Fasa Iran; ^2^ Department of Food Safety and Hygiene, School of Health Fasa University of Medical Sciences Fasa Iran; ^3^ Department of Food Sciences and Technology, School of Public Health Shahid Sadoughi University of Medical Sciences Yazd Iran; ^4^ Research Center for Food Hygiene and Safety Shahid Sadoughi University of Medical Sciences Yazd Iran

**Keywords:** bakery product, gluten‐free, inulin, quinoa

## Abstract

Diversification of gluten‐free (GF) bakery products is considerably important, as those who suffer from gluten intolerance should follow a GF diet their whole life. Regarding this study, it was aimed at optimizing the formulation of a quinoa‐based GF traditional bakery product, i.e. Nan‐e‐Fasaee using inulin as a bifunctional agent (both a prebiotic compound and a structure‐forming agent). Otherwise, its potential role as a fat and sugar replacer was also assessed. For this purpose, short (S)‐ and long (L)‐chain inulin were used as sugar and fat replacers, respectively, at 0%–50% w/w in quinoa flour (QF)‐based GF Nan‐e‐Fasaee and optimization was done based on rheological, textural, and sensory analysis. Results indicated that QF diet provided the batter with the dominance of elastic modulus and increased hardness (i.e. 5170.0 ± 22.50 g in the presence of QF compared to 1477.0 ± 20.81 g in wheat‐based ones). Inulin inclusion reduced the hardness, as the lowest was observed at S‐inulin substitution levels of 40% and 50% w/w, with values equal to 2422.0 ± 20.81 and 2431.0 ± 35.57 g, respectively (the most similar ones to control sample). The interference of S‐inulin with the non‐gelatinized starch structure is supposed to decrease the hardness. The highest score in texture was also perceived at F6 and F13, with values equal to 8.00 ± 0.10 and 7.97 ± 0.05, respectively. Using S‐ and L‐inulin in combination is found to improve the textural characteristics due to preventing the competitive role of sugar in water absorption in formulations containing L‐inulin. Regarding optimization of quinoa‐based GF Nan‐e‐Fasaee with reduced sugar and fat levels using inulin, it is found to be feasible.

## Graphical abstract

Using short‐ and long‐chain inulin (as sugar and fat substitutes, respectively) is found to improve the textural characteristics of gluten‐free (GF) Nan‐e‐Fasaee based on quinoa flour. Quinoa flour provides the batter with the dominance of elastic modulus and increased hardness, whereby its hardness is reduced by inulin inclusion. Optimization of quinoa‐based GF Nan‐e‐Fasaee with reduced sugar and fat levels using inulin is found to be feasible.

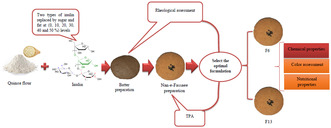

## 1 INTRODUCTION

Wheat is the main grain used in the formulation of bakery products, especially bread, because of its gluten protein. Despite the unique characteristics of gluten protein to form a three‐dimensional network, it may be a threat for some people, including celiac patients (Drabińska et al., 2016). Celiac disease (CD) is a chronic autoimmune disorder in people who are genetically sensitive to the gluten protein. Currently, approximately 1%–2% of the world population suffers from CD. Avoiding gluten consumption and following a gluten‐free (GF) diet are the only options available to control the symptoms of this disease. A long‐term GF diet can, however, lead to some issues such as digestive problems, anemia, and osteoporosis because of nutritional challenges in the GF diet associated with low protein, fiber, and mineral content (Dotsenko et al., 2021; Nestares et al., 2020). One of the main strategies to improve the nutritional value of GF products is to use nutrient‐rich flours (Nieto‐Mazzocco et al., 2020). Pseudocereals (quinoa, amaranth, and buckwheat) are valuable options for producing GF and highly nutritious foods due to their high‐quality protein, fiber, and unsaturated fatty acids (Hafez, 2018). On the other hand, pseudocereals, especially quinoa, are known as GF edible seeds with high protein content and essential nutrients with diverse medicinal and industrial uses. Thanks to their hemolytic and anti‐fat activities, these pseudocereals can reduce blood sugar and cholesterol levels. The presence of compounds with high nutritional and biological values of quinoa has further attracted the attention of food manufacturers, especially for the production of GF foods (Mir et al., 2018).

The production of GF bakery products is also limited by technological and sensorial problems due to the weak batter structure after the elimination of gluten (Sarabhai et al., 2017). In this regard, hydrocolloids are generally used as gluten mimicking agents in the formulation of GF bakery products (Cappelli et al., 2020; Culetu et al., 2021). Prebiotics are soluble fiber and indigestible compounds that can improve the function of normal flora microorganisms. They can also reduce and prevent diseases such as diabetes, osteoporosis, high cholesterol, obesity, and irritable bowel syndrome (Hutkins et al., 2016; Parnell & Reimer, 2012).

Inulin, as one of the essential and commonly used prebiotic compounds, belongs to the fructans family. It has many health effects, including enhancement of mineral absorption, anti‐cancer effects, reducing the risk of cardiovascular disease, and increasing the growth of beneficial bacteria (Gao et al., 2018; Mollakhalili‐Meybodi et al., 2021). Differences in the polymerization degree of inulin have led to the development of different types of inulin with various applications in the food and pharmaceutical industries. The short‐chain (S type) inulin is highly soluble and has a sweet taste, making it a proper substitute for sugar. The long‐chain (L type) inulin has lower solubility and can serve as a filler, introducing it as a fat substitute in low‐calorie products (Drabińska et al., 2016).

Recently, the use of pseudocereals or prebiotics in the production of functional products has aroused the minds of food industry researchers due to their special compounds. Several studies have been conducted in this field; for instance, the production of GF biscuits based on QF (Moawad et al., 2018), optimization of GF cookies using QF (Jan et al., 2018), production of prebiotic GF chocolate cookies using inulin and oligofructose (da Silva & Conti‐Silva, 2018), and the use of inulin for fat reduction in GF biscuits (Longoria‐García et al., 2020).

Nan‐e‐Fasaee is one of the most popular bakery products (in the form of cookies) with a high content of fat and sugar. This product is a type of sweet made from wheat flour, rose water, cardamom, and saffron and decorated with purslane seeds. This sweet is a souvenir of Fasa city (Fars province, Iran), and every year nearly 150 tons of Nan‐e‐Fasaee are prepared from 40 factories in this city. This sweet is crisp and dry and has a long shelf life (Institute of Standards of Iran, 1993). It has found high consumption in other cities in the country as well. This study is thus aimed at introducing an optimal formulation for GF Nan‐e‐Fasaee based on QF and inulin as a functional gluten mimicking agent, whose characteristic of reducing the sugar and fat content of the final product is also appealing to be monitored. In this regard, S‐ and L‐chain‐inulin are used in formulation as sugar and fat replacement, respectively, and its potential to improve technological characteristics is monitored by the rheological, textural, sensorial, and nutritional characteristics of GF Nan‐e‐Fasaee based on QF.

## 2 MATERIALS AND METHODS

### 2.1 Raw materials

Wheat flour (WF) (contained moisture, ash, and fat at 13.72%, 1.29%, and 1.01% w/w, respectively) was purchased from a local market (Fasa, Iran). QF (contained moisture, ash, and fat at 11.03%, 1.37%, and 2.74% w/w, respectively) was also obtained from Razavi Qods Company. Inulin (short‐chain (S) (Frutalose OFP, inulin content≥92%, DP≤10), long‐chain (L) (Frutaft TEX, inulin content≥99.5% DP≥23)) was supplied from SENSUS, and oil (solid and liquid) was provided from Varamin Company. Other necessary ingredients like sugar and vinegar were bought from a local market. All of the chemicals (necessary for physicochemical analysis) were purchased from Merck Company, Germany.

### 2.2 Sample preparation

Wheat or QF (500 g), sugar syrup (200 g), solid oil (250 g), and liquid oil (50 g) were used in sample preparation. In a typical procedure, 250 g of pure confectionery oil was mixed by a home mixer (Dessini 202) at medium speed for 10 min. Then, 250 g of flour was added and mixed with the oil. Other ingredients (including another 250 g of flour, 50 g of liquid oil, and 200 g of syrup sugar) were then gradually added to the previous mixture and mixed for 2 min to obtain a uniform batter. S‐chain and L‐chain inulin as sugar and fat replacement, respectively, were added to the batter at 6 levels (i.e. 0%, 10%, 20%, 30%, 40%, and 50% w/w). The resulting batters were placed in plastic bags and kept in the refrigerator for 24 h. Table 1 shows the formulation of the prepared batters. For the preparation of the final samples, all prepared batters were kneaded by hand for 30 min and spread on a tray with a rolling pin. The batter was then cut into circles of 5 cm in diameter using metal molds. Finally, the formed and sliced batters were placed in a preheated oven (Sharp) at 130°C for 20 min.

**TABLE 1:** Formulations of gluten‐free Nan‐e‐Fasaee samples based on quinoa flour and inulin.

Number	Type of flour	Sugar and S‐inulin ratio		Fat and L‐inulin ratio		Formulation
		Sugar	S‐chain inulin	Fat	L‐chain inulin	
1	W	100	0	100	0	F1 (Control)
2	Q	100	0	100	0	F2
3	Q	90	10	100	0	F3
4	Q	80	20	100	0	F4
5	Q	70	30	100	0	F5
6	Q	60	40	100	0	F6
7	Q	50	50	100	0	F7
8	Q	100	0	90	10	F8
9	Q	100	0	80	20	F9
10	Q	90	10	90	10	F10
11	Q	90	10	80	20	F11
12	Q	80	20	90	10	F12
13	Q	70	30	90	10	F13
14	Q	70	30	80	20	F14
15	Q	60	40	90	10	F15
16	Q	50	50	90	10	F16
17	Q	100	0	70	30	NDF
18	Q	100	0	60	40	NDF
19	Q	100	0	50	50	NDF
20	Q	90	10	70	70	NDF
21	Q	90	10	60	40	NDF
22	Q	90	10	50	50	NDF
23	Q	80	20	80	20	NDF
24	Q	80	20	70	30	NDF
25	Q	80	20	70	30	NDF
26	Q	80	20	60	40	NDF
27	Q	70	30	70	30	NDF
28	Q	70	30	60	40	NDF
29	Q	70	30	50	50	NDF
30	Q	60	40	80	20	NDF
31	Q	60	40	70	30	NDF
32	Q	60	40	60	40	NDF
33	Q	60	40	50	50	NDF
34	Q	50	50	80	20	NDF
35	Q	50	50	70	30	NDF
36	Q	50	50	60	40	NDF
37	Q	50	50	50	50	NDF

Abbreviations: L, long chain inulin; NDF, No dough formation; Q, quinoa flour; S, short chain inulin; W, wheat flour.

### 2.3 Rheological properties of batter

#### 2.3.1 Frequency sweep test

The frequency sweep test was carried out by a rheometer equipped with a parallel plate geometer (with a probe diameter of 2.5 cm) at constant temperature of 25°C (Antonpar Company). The frequency dependence of storage modulus (G′) and loss modulus (G′′) was evaluated in the frequency range of 0.1–1.0 Hz and constant strain of 0.01 according to Moriano et al. (2018). The damping factor (tan δ) was determined by Equation 1.

(1) $$\tan\delta =\frac{G^{\prime \prime }}{G^{\prime }}$$

#### 2.3.2 Temperature sweep test

A temperature sweep test was also performed by a rheometer (Anton par Company) in the temperature range of 25–100°C with a heating rate of 5°C/min. G′ and G′′ were recorded at a frequency and strain of 1 Hz and 0.05%, respectively (Zhang et al., 2017). The tan (δ) was also recorded during the temperature sweep, according to Equation 1.

### 2.4 Texture analysis

The textural characteristics of the Nan‐e‐Fasaee samples were determined by a texture analyzer (CT3 4500). An aluminum cylindrical probe (25 mm diameter) was used in a Texture Profile Analyzer (TPA) by double compression test to penetrate up to 50% depth at a rate of 2 mm/s. The delay between the first and second compressions was 30 s (Mohammadi et al., 2021). Hardness was a parameter that was obtained from the TPA of the samples.

### 2.5 Sensory analysis

The Nan‐e‐Fasaee samples prepared in this study were assessed by 50 trained panelists from members (30 students +20 staff) of Fasa University of Medical Science and evaluated according to texture, taste, aroma, color, mouthfeel, and overall acceptability based on the 9‐point hedonic scale method, in which scores of 1, 5, and 9 indicated very unpleasant, acceptable, and extremely pleasant, respectively. For this aim, the encoded Nan‐e‐Fasaee samples were distributed among the panelists with water that was served before the testing of each sample (Mohammadi et al., 2021). Results are represented in Table 2.

**TABLE 2:** Results of sensory analysis for gluten‐free Nan‐e‐Fasaee based on quinoa flour and inulin.

Samples***	Sensory paramete					
	Texture*	Taste	Aroma	Color	Mouthfeel	Overall acceptance
F1	8.57 ± 0.09^a^ **	8.35 ± 0.03^a^	7.64 ± 2.06^a^	7.57 ± 1.91^a^	7.78 ± 1.06^a^	8.85 ± 0.46^a^
F2	7.00 ± 0.06^e^	7.48 ± 0.08^b^	7.28 ± 1.58^a^	7.21 ± 1.08^a^	7.21 ± 1.47^a^	7.07 ± 0.32^d^
F3	7.19 ± 0.04^d^	7.41 ± 0.06^b^	6.50 ± 2.47^a^	7.50 ± 1.67^a^	7.50 ± 1.34^a^	7.50 ± 0.50^c^
F4	7.31 ± 0.01^d^	7.35 ± 0.08^b^	7.28 ± 2.43^a^	7.14 ± 2.21^a^	7.14 ± 1.65^a^	7.57 ± 0.78^c^
F5	7.57 ± 0.05^c^	7.21 ± 0.08^b^	7.21 ± 2.25^a^	7.21 ± 2.29^a^	7.21 ± 1.42^a^	7.21 ± 0.44^d^
F6	8.00 ± 0.10^b^	7.36 ± 0.01^b^	7.00 ± 1.88^a^	7.35 ± 1.94^a^	7.50 ± 1.22^a^	8.28 ± 0.32^b^
F7	7.51 ± 0.03^c^	7.50 ± 0.02^b^	7.28 ± 2.23^a^	7.28 ± 2.01^a^	7.42 ± 1.45^a^	7.85 ± 0.56b^c^
F8	6.21 ± 0.12^f^	7.07 ± 0.07^bc^	6.92 ± 2.23^a^	7.57 ± 2.10 ^a^	6.85 ± 1.87^a^	6.78 ± 0.71^e^
F9	7.00 ± 0.07^d^	6.57 ± 0.03^c^	7.07 ± 1.81^a^	7.21 ± 1.84^a^	7.14 ± 1.40^a^	6.78 ± 0.18^e^
F10	5.85 ± 0.09^g^	6.50 ± 0.05^c^	7.21 ± 1.67^a^	6.92 ± 1.89^a^	6.85 ± 1.65^a^	7.00 ± 0.46^d^
F11	6.85 ± 0.11^e^	6.48 ± 0.06^c^	6.78 ± 2.11^a^	7.07 ± 1.73^a^	7.07 ± 1.68^a^	6.92 ± 0.14^e^
F12	7.00 ± 0.06^e^	6.50 ± 0.01^c^	7.21 ± 1.96^a^	7.14 ± 2.21^a^	6.92 ± 2.05^a^	7.06 ± 0.01^d^
F13	7.97 ± 0.05^b^	7.25 ± 0.04^b^	7.07 ± 1.77^a^	6.92 ± 2.12^a^	6.71 ± 1.48^a^	8.08 ± 0.11^b^
F14	7.78 ± 0.01^b^	7.18 ± 0.05^b^	7.35 ± 1.82^a^	6.92 ± 2.12^a^	6.71 ± 1.38^a^	7.58 ± 0.20^bc^
F15	7.64 ± 0.03^bc^	6.57 ± 0.03^c^	7.00 ± 2.14^a^	7.35 ± 1.86^a^	7.00 ± 2.14^a^	7.14 ± 0.07^de^
F16	7.07 ± 0.07^e^	6.58 ± 0.08^c^	7.14 ± 2.24^a^	7.14 ± 2.07 ^a^	7.21 ± 2.71^a^	7.28 ± 0.63^d^

* Data are reported as average ± standard deviation.

** The different letters in each column show the significant difference (*p* < .05).

*** F1 = control sample based on WF, F2 = gluten‐free sample based on QF without inulin, F3 = gluten‐free sample based on QF and 10% w/w S‐inulin, F4 = gluten‐free sample based on QF and 20% w/w S‐inulin,F5 = gluten‐free sample based on QF and 30% w/w S‐inulin, F6 = gluten‐free sample based on QF and 40% w/w S‐inulin, F7 = gluten‐free sample based on QF and 50% w/w S‐inulin, F8 = gluten‐free sample based on QF and 10% w/w L‐inulin, F9 = gluten‐free sample based on QF and 20% w/w L‐inulin, F10 = gluten‐free sample based on QF and (10% + 10%)w/w S‐ and L‐inulin, F11 = gluten‐free sample based on QF and (10% + 20%)w/w S‐ and L‐inulin, F12 = gluten‐free sample based on QF and (20% + 10%)w/w S‐ and L‐inulin, F13 = gluten‐free sample based on QF and (30% + 10%)w/w S‐ and L‐inulin, F14 = gluten‐free sample based on QF and (30% + 20%)w/w S‐ and L‐inulin, F15 = gluten‐free sample based on QF and (40% + 10%)w/w S‐ and L‐inulin, F16 = gluten‐free sample based on QF and (50% + 10%)w/w S‐ and L‐inulin.

### 2.6 Analysis of optimal GF Nan‐e‐Fasaee samples

Samples with preferred rheological and textural characteristics, as well as better sensory evaluation results, were selected as the optimal GF prebiotic Nan‐e‐Fasaee samples. Some of their physicochemical and nutritional characteristics were measured and compared with the control samples (F1 and F2). Table 3 shows the results of optimal samples.

**TABLE 3:** Results of chemical analysis for optimal gluten‐free Nan‐e‐Fasaee based on quinoa flour and inulin.

Samples**	Moisture (%)	Chemical parameters					
		Ash (%)	Protein (%)	Fat (%)	Fiber (%)	Carbohydrate (%)	Calorie value
F1	0.87 ± 0.05^c^ *	0.33 ± 0.0^b^	6.9 ± 0.04^b^	33.36 ± 0.04^a^	0.4 ± 0.04^c^	58.54 ± 0.05^a^	562 ± 0.04^a^
F2	3.07 ± 0.05^a^	1.12 ± 0.05 ^a^	10.2 ± 0.05^a^	32.5 ± 0.05^c^	1.53 ± 0.05^a^	53.11 ± 0.05^c^	545.72 ± 0.04^b^
F6	3.04 ± 0.04^a^	1.09 ± 0.05^a^	10.2 ± 0.05^a^	32.9 ± 0.05^b^	1.11 ± 0.05^b^	52.77 ± 0.05^d^	537.98 ± 0.03^c^
F13	2.08 ± 0.03^b^	1.10 ± 0.05^a^	10.3 ± 0.05^a^	29 ± 0.05^d^	1.15 ± 0.05^b^	56.72 ± 0.05^d^	529.08 ± 0.04^d^

*Note*: Data are reported as average ± standard deviation.

* The different letters in each column show the significant difference (*p* < .05).

** F1 = control sample based on WF, F2 = gluten‐free sample based on QF without inulin, F6 = optimal gluten‐free sample based on QF and 40% w/w S‐inulin, F13 = optimal gluten‐free sample based on QF and (30% + 10%)w/w S‐ and L‐inulin.

#### 2.6.1 Chemical analysis

The moisture, ash, protein, total fat, and crude fiber contents of optimal GF prebiotic Nan‐e‐Fasaee samples were measured according to the AOAC (AOAC, 1990) method. Carbohydrate content was measured according to Equation 2 (Agu & Okoli, 2014).

(2) $$\text{Carbohydrate}\%=100–\left(\text{moisture}\%+\text{protein}\%+\mathrm{fat}\%+\mathrm{ash}\%+\text{crude fiber}\%\right).$$

The calorie of optimal sample was also calculated by Equation 3. (Farzana & Mohajan, 2015).

(3) $$\text{Calorie value}\;\left(\mathrm{Cal}/100g\;\text{of sample}\right)=4\;\left(\text{carbohydrate}\;+\text{protein}\right)\%+9\;\left(\mathrm{fat}\%\right).$$

#### 2.6.2 Color analysis

The surface color of optimal GF prebiotic Nan‐e‐Fasaee samples was measured by a Hunter lab calorimeter (Hunter lab D25). The results were reported in L*, a*, and b* values corresponding to brightness, redness, and yellowness, respectively (Mohammadi et al., 2021).

### 2.7 Statistical analysis

Statistical analysis was performed by SPSS V.18 software. A one‐way ANOVA was utilized for evaluating the statistical significance, while the difference between the samples was assessed by the Tukey's test at a significance level of 5% (*p* < .05). All the tests were carried out in triplicate, and results were reported based on the mean and standard deviation (mean ± SD).

## 3 RESULTS AND DISCUSSION

In general, among the 36 different formulations of GF batters, just 15 uniform batter formulations were obtained. Therefore, 16 different batters (15 GF samples + control) were made and labeled F1 to F16 as listed in Table 1.

### 3.1 Rheological characteristics

#### 3.1.1 Frequency sweep test

**FIGURE 1:**
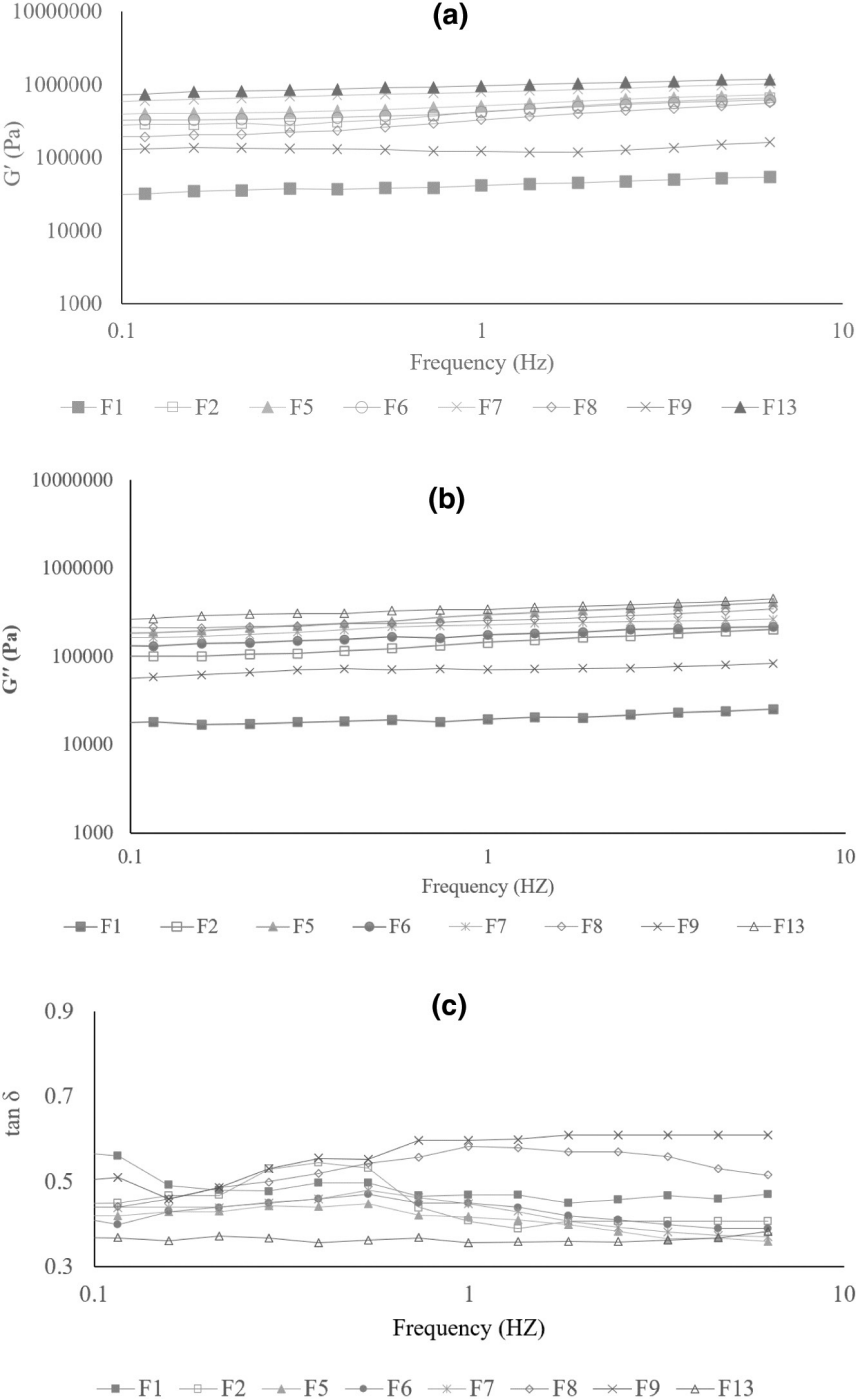
(a–c). Frequency sweep curves of GF Nan‐e‐Fasaee batters (curves are the average of triplicate): (a) is storage modulus (G′), (b) is loss modulus (G′′), and (c) is damping factor (tan δ). F1 = control sample based on WF, F2 = gluten‐free sample based on QF without inulin, F5 = gluten‐free sample based on QF and 30% w/w S‐inulin, F6 = gluten‐free sample based on QF and 40% w/w S‐inulin, F7 = gluten‐free sample based on QF and 50% w/w S‐inulin, F8 = gluten‐free sample based on QF and 10% w/w L‐inulin, F9 = gluten‐free sample based on QF and 20% w/w L‐inulin, F13 = gluten‐free sample based on QF and (30% + 10%)w/w S‐ and L‐inulin.

The frequency sweep test was performed to investigate the impact of formulation on the viscoelastic characteristics of batter in terms of processability and quality of the final product by providing storage modulus (G′), loss modulus (G′′), and damping factor (tanδ) (Han et al., 2019; Witczak et al., 2017). The frequency sweep tests of different formulations of Nan‐e‐Fasaee samples in this study are shown in Figure 1a–c (however, the rheological behavior of all 16 samples is assessed; the graphs with an obvious trend are only shown to make them more understandable).

As depicted in Figure 1a–c, all GF formulations had a greater elastic modulus compared to viscose modulus (tan δ < 1), confirming the formation of gel‐like structures in GF batter (Mollakhalili Meybodi et al., 2015; Shiri et al., 2021). However, an increased G' and G" have been observed at the GF formulation in the absence of inulin (F2) compared to the wheat‐based formulation (F1). No uniform trend has been found in the presence of inulin. Previously, it has been stated that the presence of inulin decreased the viscoelastic behavior in a polymerization degree‐dependent manner (Matos & Rosell, 2015).

However, no uniform trend was observed on frequency dependency of G′ and G′′ moduli by gradual reduction of sugar by replacement of S‐inulin with similar fat content (i.e. F3–F7). Elastic modulus has been increased at formulations containing S‐inulin at 30%, 40%, and 50% w/w (F5, F6, and F7, respectively) which is attributed to reduction of the lubricating role of sugar (Blanco Canalis et al., 2017).

Considering the impact of fat replacement by L‐inulin, it seems that fat plays a critical role in Nan‐e‐fasaee formulation, as its replacement at levels higher than 20% w/w did not lead to dough formation. At a constant level of sugar (sugar:inulin ratio of 100:0), by incorporation of L‐inulin at levels of 10% and 20% w/w (i.e. F8 and F9, respectively), a decrease in viscous modulus and an increase in elastic modulus have been found, which is in accordance with (Krystyjan et al., 2015).

#### 3.1.2 Temperature sweep test

**FIGURE 2:**
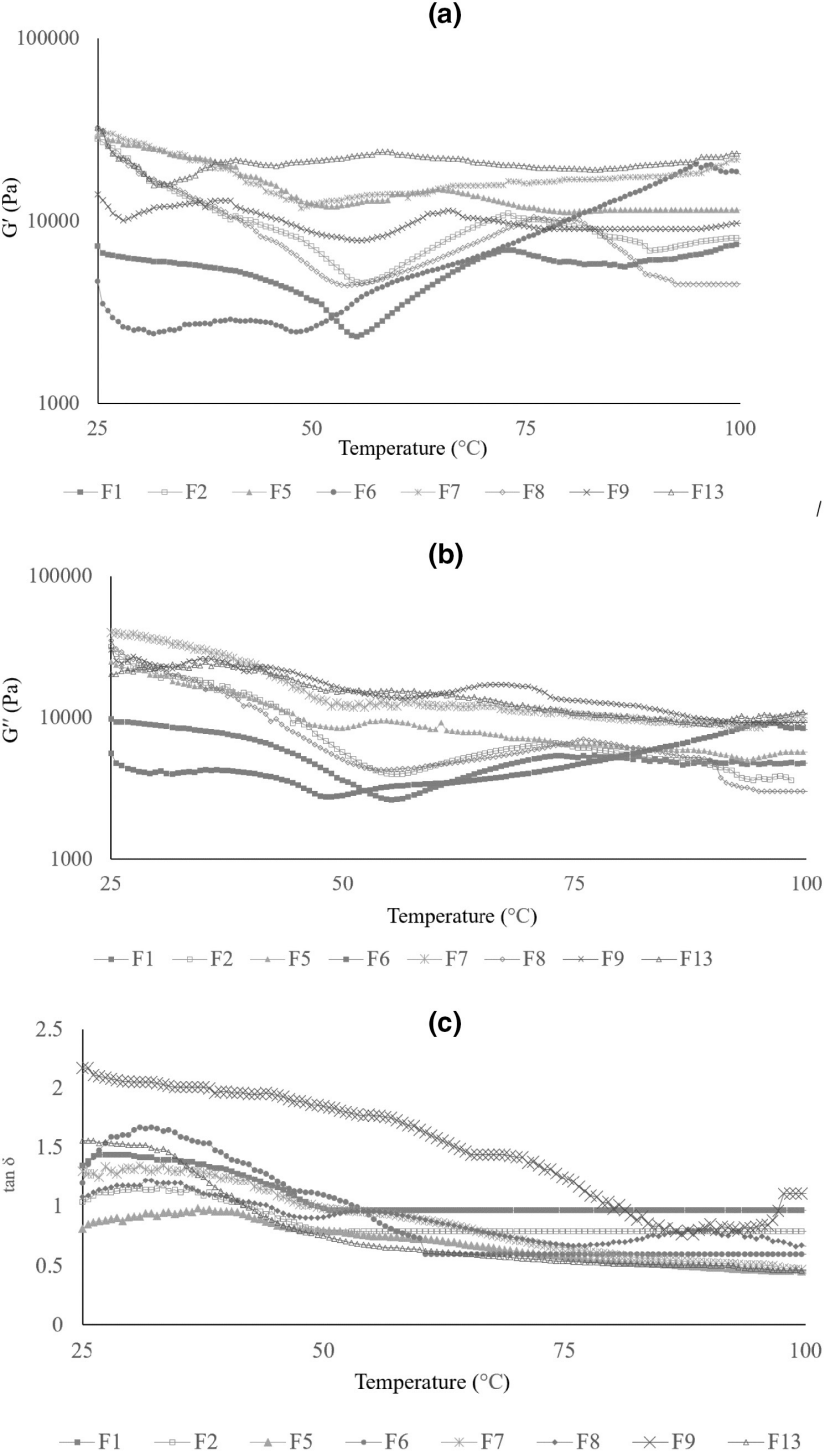
(a–c). Temperature sweep curves of GF Nan‐e‐Fasaee batters (curves are the average of triplicate): (a) is storage modulus (G′), (b) is loss modulus (G′′), and (c) is damping factor (tan δ). F1 = control sample based on WF, F2 = gluten‐free sample based on QF without inulin, F5 = gluten‐free sample based on QF and 30% w/w S‐inulin, F6 = gluten‐free sample based on QF and 40% w/w S‐inulin, F7 = gluten‐free sample based on QF and 50% w/w S‐inulin, F8 = gluten‐free sample based on QF and 10% w/w L‐inulin, F9 = gluten‐free sample based on QF and 20% w/w L‐inulin, F13 = gluten‐free sample based on QF and (30% + 10%)w/w S‐ and L‐inulin.

Alongside the viscoelastic characteristics of the raw batter, the structural changes that occur through baking are also investigated to determine its stability during the development of the final product (Sanz et al., 2008). Regarding the temperature sweep test of GF Nan‐e‐Fasaee samples, it was determined as presented in Figure 2a–c. As illustrated, when the temperature reached the starch gelatinization temperature, both G' and G“ started to increase (this temperature was similar for both wheat‐based and quinoa‐based formulations in the absence of inulin (i.e. F1 and F2, respectively) at around 53.5°C). However, at the quinoa‐based one (i.e. F2), both G' and G" will then start to decrease at around 70°C. The results also illustrated that the enhancement of viscous and elastic moduli in samples with higher substitution levels of sugar by S‐inulin began at a lower temperature (about 50°C). In other words, it seems that S‐inulin incorporation reduced the gelatinization temperature of starch in formulations, which is attributed to their lower sugar contents. This result is in accordance with (Laguna et al., 2013; Struck et al., 2016; Tsatsaragkou et al., 2021).

The presence of inulin at formulation changes the starch gelatinization behavior according to their polymerization degree, substitution level, and the way they are incorporated either as a fat or sugar replacer.

The temperature dependency of the damping factor (tan δ) as a representative of the proportion of viscose and elastic components of formulations is demonstrated in Figure 2c. A similar pattern has been found for wheat and quinoa‐based formulations (i.e. F1 and F2). However, the tan δ is higher for wheat‐based ones, which means a higher dominance of viscose behavior in wheat‐based formulations, which can be attributed to the higher protein content of QF compared to WF (Bozdogan et al., 2019; Li & Zhu, 2018). The highest damping factor is found at F9, which contains L‐type inulin as a fat replacer at 20% w/w, which is attributed to the L‐inulin ability to trap water at a higher substitution level, which reduces elastic properties (Blanco Canalis et al., 2017).

Four stages can be considered for tan δ development during heating. In other words, at first, it decreased in both samples at around 50°C, which can be attributed to the starch fraction, which absorbed the available free water and swelled (Kumar et al., 2018). Then it has been increased by heating up to 55°C, which is attributed to the dissolution of sugar as well as the melting of fat (Blanco Canalis et al., 2017; Zhang et al., 2017). It has then been decreased again by heating up to 75°C, which can be attributed to the gelatinization of starch (Onyango et al., 2011). In other words, by increasing the temperature, the protein structure was denatured, and the remaining starch particles absorbed the available free water and swelled. Finally, the elasticity and hardness of the batters will be enhanced. The increase in tan δ through heating at around 75–88°C is supposed to be induced by the breaking of starch granules by reaching their highest swelling state (Bozdogan et al., 2019; Xu et al., 2020).

Generally, S‐inulin incorporation at levels of 10%, 20%, and 30% w/w in F3, F4, and F5 samples, respectively, provided batters with higher G′ and G′′ and lower tan (δ). In other words, S‐inulin incorporation at these ratios could strengthen the batter structure by binding water and forming a more solid‐like structure (Kim et al., 2019).

### 3.2 Texture evaluation

**FIGURE 3:**
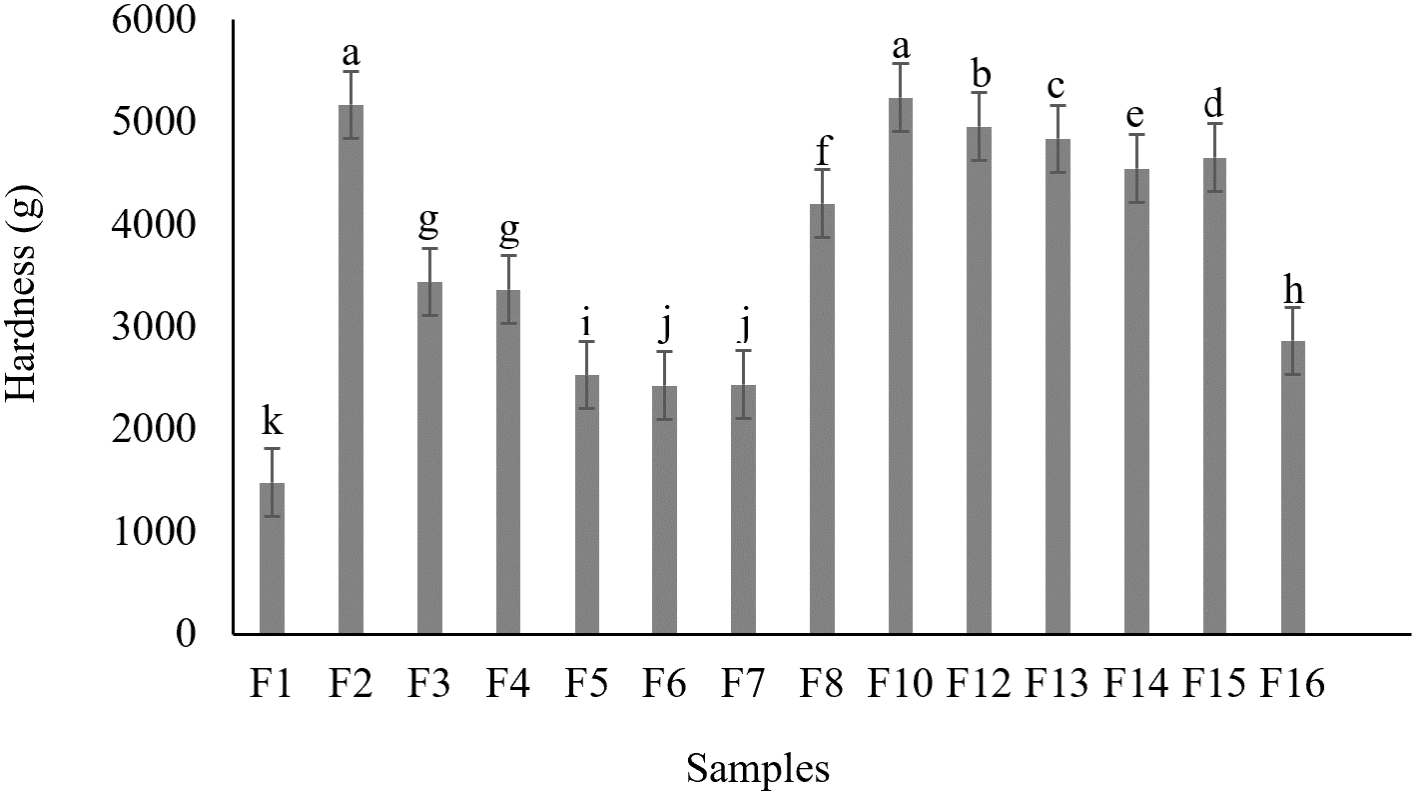
The appearance of the final gluten‐free Nan‐e‐Fasaee based on quinoa flour and inulin after cooking. F1 = control sample based on WF, F2 = gluten‐free sample based on QF without inulin, F3 = gluten‐free sample based on QF and 10% w/w S‐inulin, F4 = gluten‐free sample based on QF and 20% w/w S‐inulin, F5 = gluten‐free sample based on QF and 30% w/w S‐inulin, F6 = gluten‐free sample based on QF and 40% w/w S‐inulin, F7 = gluten‐free sample based on QF and 50% w/w S‐inulin, F8 = gluten‐free sample based on QF and 10% w/w L‐inulin, F9 = gluten‐free sample based on QF and 20% w/w L‐inulin, F10 = gluten‐free sample base on QF and (10% + 10%)w/w S‐ and L‐inulin, F11 = gluten‐free sample based on QF and (10% + 20%)w/w S‐ and L‐inulin, F12 = gluten‐free sample based on QF and (20% + 10%)w/w S‐ and L‐inulin, F13 = gluten‐free sample based on QF and (30% + 10%)w/w S‐ and L‐inulin, F14 = gluten‐free sample based on QF and (30% + 20%)w/w S‐ and L‐inulin, F15 = gluten‐free sample based on QF and (40% + 10%)w/w S‐ and L‐inulin, F16 = gluten‐free sample based on QF and (50% + 10%)w/w S‐ and L‐inulin.

**FIGURE 4:**
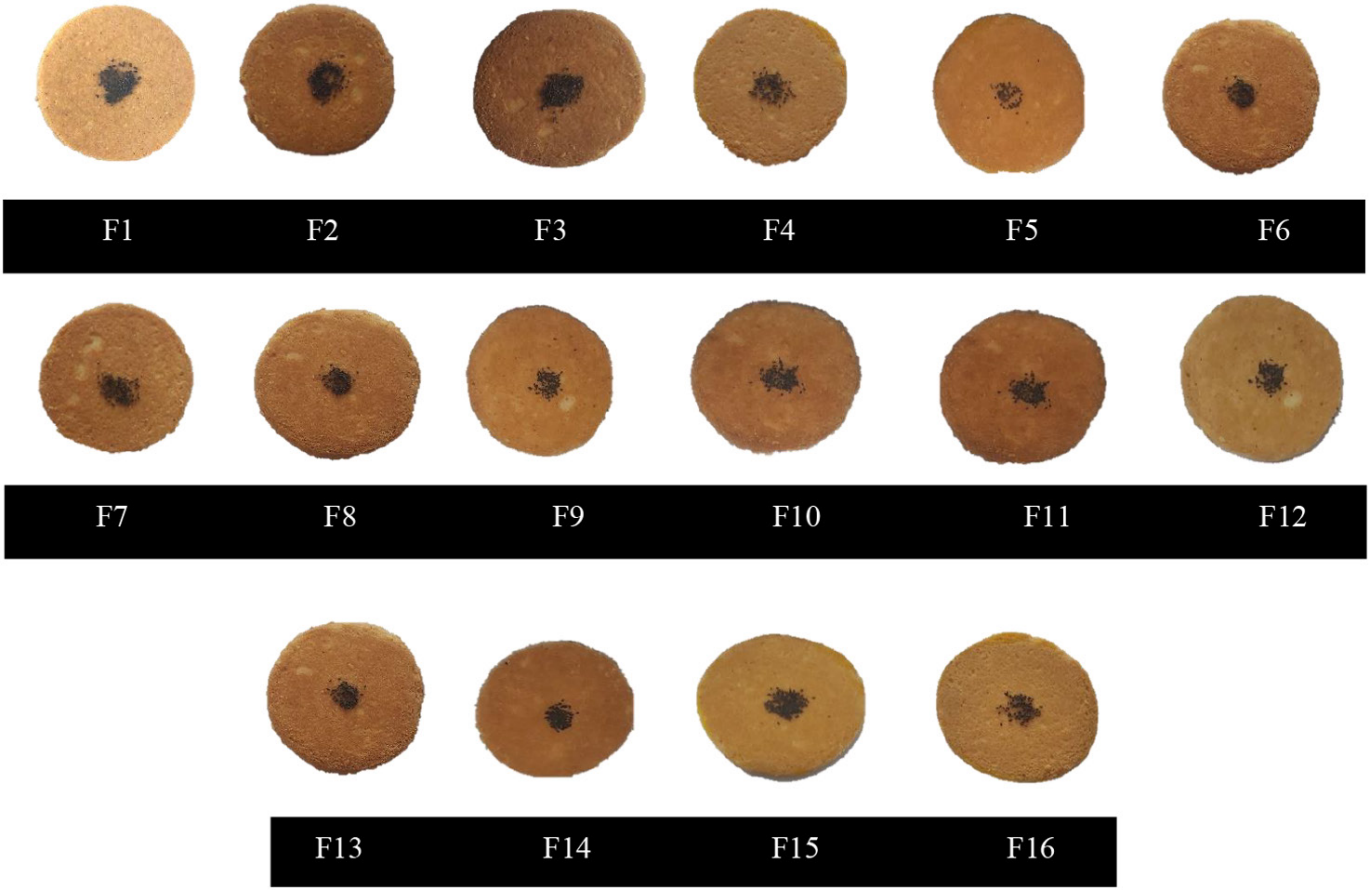
Results of TPA (Hardness) for gluten‐free Nan‐e‐Fasaee based on QF and inulin. F1 = control sample based on WF, F2 = gluten‐free sample based on QF without inulin, F3 = gluten‐free sample based on QF and 10% w/w S‐inulin, F4 = gluten‐free sample based on QF and 20% w/w S‐inulin, F5 = gluten‐free sample based on QF and 30% w/w S‐inulin, F6 = gluten‐free sample based on QF and 40% w/w S‐inulin, F7 = gluten‐free sample based on QF and 50% w/w S‐inulin, F8 = gluten‐free sample based on QF and 10% w/w L‐inulin, F9 = gluten‐free sample based on QF and 20% w/w L‐inulin, F10 = gluten‐free sample base on QF and (10% + 10%)w/w S‐ and L‐inulin, F11 = gluten‐free sample based on QF and (10% + 20%)w/w S‐ and L‐inulin, F12 = gluten‐free sample based on QF and (20% + 10%)w/w S‐ and L‐inulin, F13 = gluten‐free sample based on QF and (30% + 10%)w/w S‐ and L‐inulin, F14 = gluten‐free sample based on QF and (30% + 20%)w/w S‐ and L‐inulin, F15 = gluten‐free sample based on QF and (40% + 10%)w/w S‐ and L‐inulin, F16 = gluten‐free sample based on QF and (50% + 10%)w/w S‐ and L‐inulin.

The textural characteristics of GF Nan‐e‐Fasaee samples containing quinoa and two types of inulin (as represented in Figure 3) are represented in Figure 4. The TPA characteristics of the F9 and F11 samples were not recorded due to the absence of their data because of their high hardness. The impact of L‐inulin incorporation on hardness has been observed to be dependent on the sugar level. In other words, despite no products obtained at formulations containing a high level of sugar, they have been improved at those containing a higher level of short‐chain inulin, which is supposed to be induced by the competitive role of sugar in water absorption and the prevention of structure formation.

As illustrated in Figure 4, the lowest and highest hardness were found in F1 (control) and F10 (GF sample containing S‐ and L‐inulin at 10% w/w), with quantities equal to 1477.20 ± 0.81 and 5240.0 ± 30.11 g, respectively. Incorporation of QF in formulations of Nan‐e‐Fasaee is found to significantly increase the hardness of GF samples compared to the control (*p < .05*). The increase in hardness of QF samples is attributed to the absence of a gluten network and their lower gelatinization of starch, which is induced by the higher protein and fiber content of QF (Mousa, 2022; Qin et al., 2022). This finding, which is also verified by rheological tests, is in accordance with Altındağ et al. (2015)).

Despite the increase observed in the hardness of GF samples, it was again decreased by inulin incorporation, depending on inulin DP and the way it is incorporated either as a fat or sugar replacer. In other words, the higher hardness of the GF sample (i.e. F2) is attributed to its weaker structure and water migration in the formulation, which were reduced and improved by inulin incorporation as a structure‐creating factor (Xu et al., 2020).

The inulin type and substitution level were assessed as two important factors in improving the hardness of GF samples. In fact, substitution with S‐inulin led to a greater reduction in hardness compared to GF samples containing L‐inulin. Moreover, the higher levels of S‐inulin significantly reduced the hardness of the samples (*p < .05*). Among GF samples, the lowest hardness was observed in samples with S‐inulin substitution levels of 40 and 50% w/w (i.e. F6 and F7, respectively), which were found to be the most similar to the control sample (F1). Therefore, the reduced crystallization rate of cooked samples after cooling can be attributed to the decremented sugar content and its replacement with S‐inulin, which interferes with the structure of non‐gelatinized starch and thus leads to a decrease in hardness (Kiumarsi et al., 2019). The GF samples with L‐inulin (i.e. F8 and F9) had higher hardness than those containing S‐inulin. As declared in Figure 4, the increased hardness induced by L‐inulin incorporation is alleviated by increasing the portion of S‐inulin. Considering the synergistic softening effect of fat and the reduction of water availability for starch and other hydrophilic compounds, fat replacement by L‐inulin will eventually cause a harder texture in the final low‐fat product (Emami et al., 2018).

### 3.3 Sensory evaluation

Sensory evaluation as a quality determinative factor plays an important role in the development of new formulations of food products as it considers consumer behavior in the overall acceptability determination of the final product. So the sensory evaluation of GF prebiotic Nan‐e‐Fasaee samples in terms of texture, taste, aroma, color, mouthfeel, and overall acceptability was done, and represented in Table 2.

As indicated in Table 2, all GF samples received a score higher than 5 (i.e. acceptable) in all parameters in sensory evaluation. Considering the parameters investigated, aroma, color, and mouthfeel could not clearly differentiate between samples. The parameters regarding taste, texture, and overall acceptability are considered in next.

In inulin‐containing formulations, the highest score in texture was perceived at F6 (containing S‐inulin at 40% w/w) and F13 (containing the complex of S‐ and L‐inulin at levels of 30% and 10% w/w, respectively), with values equal to 8.00 ± 0.10 and 7.97 ± 0.05, respectively. The higher texture perception of F6 is supposed to be influenced by its soft texture. Using short and long chains in combination is also found to improve the textural characteristics, which is attributed to preventing the competitive role of sugar in water absorption in formulations containing L‐inulin. As mentioned before, the higher textural perception of F6 can be attributed to its lower hardness, which is supposed to be induced by its lower crystallization rate after cooling through its decremented sugar content and its replacement with S‐inulin which interferes with the structure of non‐gelatinized starch (Kiumarsi et al., 2019).

Generally, all GF samples had a mild bitter aftertaste, which is related to the presence of saponin and phenolic compounds in QF (Cannas et al., 2020) which, of course, was not annoying for consumers. However, its intensity was different at different formulations. In fact, S‐inulin incorporation is found to not significantly reduce the taste perception by the consumer even at its highest, which is attributed to its hiding impacts on the bitter taste of QF, increasing the sweet taste; also, due to the decrease in hardness due to the decrease in the sugar level as a factor preventing the gelatinization of starch, these samples had a suitable texture too (Kiumarsi et al., 2019).

Overall acceptance is considered the main determinative parameter in consumer perception of the new formulation (Mohammadi et al., 2021). Results indicated the highest score was found at F6 and F13, with values equal to 8.28 ± 0.32 and 8.08 ± 0.11, respectively. Correlation analysis of sensory characteristics and instrumental measurement through this study have illustrated that the scores for overall acceptance were significantly and positively correlated with texture and taste (*r* = .82 and .782, respectively, *p* ≤ 0.05). F6 and F13 are considered as the optimized GF formulations, whose characterizations are summarized below.

So because of the importance of these two indicators to the production of Nan‐e‐Fasaee, F6 and F13 samples were considered as the optimal samples in a recent study.

### 3.4 Evaluation of optimal samples

Considering the average of rheological, textural, and sensorial results of GF Nan‐e‐Fasaee samples, two samples had the desirable characteristics and were selected as the optimal GF prebiotic Nan‐e‐Fasaee samples. These samples were F6 (containing S‐inulin at a level of 40% w/w) and F13 (containing the complex of S‐ and L‐inulin at levels of 30% and 10% w/w, respectively). After the selection of optimal samples, their chemical characteristics, color, and antioxidant activity were evaluated and compared with control samples, as represented in Tables 3 and 4.

**TABLE 4:** Color analysis of optimal gluten‐free Nan‐e‐Fasaee based on quinoa flour and inulin.

Samples**	Color value		
	L*	a*	b*
F1	73.33 ± 4.27^a*^	4.83 ± 0.40^a^ *	27.00 ± 7.37^a^
F2	42.03 ± 3.89^b^	1.33 ± 0.81^b^	23.50 ± 1.64^a^
F6	43.00 ± 1.73^b^	1.57 ± 0.47^b^	20.57 ± 3.82^a^
F13	43.85 ± 2.85^b^	1.28 ± 0.40^b^	21.85 ± 1.71^a^

*Note*: L*, a* and b* correspond to brightness, redness and yellowness respectively.

* The different letters in each column show the significant difference (*p* < .05).

** F1 = control sample based on WF, F2 = gluten‐free sample based on QF without inulin, F6 = optimal gluten‐free sample based on QF and 40% w/w S‐inulin, F13 = optimal gluten‐free sample based on QF and (30% + 10%)w/w S‐ and L‐inulin.

#### 3.4.1 Chemical evaluation

The characterization of optimal and control samples was done and reported as moisture, ash, protein, fat, fiber, carbohydrate, and calorie value quantification. As depicted in Table 3, despite an increase in the moisture content of GF samples, which is in accordance with Kaur et al. (2015)), it has decreased significantly (*p < .05)* at F13, which is attributed to its well‐organized structure, which facilitates water migration through the baking process (Mohammadi et al., 2021; Shiri et al., 2021).

All GF samples show significantly higher total ash and protein contents (*p* < .05), as the lowest content of ash and protein was measured in the control sample at 0.33 ± 0.04 and 6.9 ± 0.04 percent, respectively. The high content of crude ash and protein in GF samples is one of the desirable characteristics in terms of nutritional value and mineral content, as confirmed by Chiş et al. (2020)) who investigated the effect of quinoa in GF muffins.

As illustrated in Table 3, the highest fat content was observed in the control sample (i.e. F1) at 33.36 ± 0.04 percent (*p* < .05), which is related to the lower fat content of QF compared to WF. A similar result was also reported by Xu et al. (2020)) in studying the effect of QF addition on wheat bread. The fat content of F6 is found to be significantly higher than that of F13 (*p* < .05), which was due to the substitution of L‐inulin with fat at a level of 10% w/w in the formulation of the F13 sample (Longoria‐García et al., 2020).

Considering the importance of fiber content in the nutritional characterization of GF Nan‐e‐Fasaee, its assessment has also been done. As illustrated in Table 3, the fiber content in the evaluated samples was in the range of 0.4%–1.53%; the lowest value was assigned to control sample at 0.4 ± 0.04%. In other words, QF incorporation significantly enhanced the fiber content of optimal samples (*p < .05*). The increased fiber content in the optimal sample is attributed to the higher fiber content in QF (Wang et al., 2021).

As illustrated in Table 3, the highest content of carbohydrate was found at F1 (i.e. control) at a value of 58.54 ± 0.05%. The lowest content of carbohydrate was found in the F6 sample (optimal sample containing S‐inulin at a level of 40% w/w), which is attributed to the lower level of sugar in this formulation.

Results was demonstrated that the highest calorie value was found in the control sample (i.e. F1) at 562 ± 0.04 Kcal/100 g, with simultaneously the lowest fiber content and the highest content of fat and carbohydrates. In other words, QF and two types of inulin incorporation in optimal samples caused a significant decrease in calorie value compared to the control sample (*p* < .05). Also, Drakos et al. (2021)) confirmed the direct correlation of reduced sugar and fat content and calorie value.

Since the aim of most studies investigating the production of GF confectionery products is to reduce the calorie intake, it seems that the optimal GF Nan‐e‐Fasaee samples in this study have the potential to be introduced as a sweet product with reduced calories. Additionally, the optimal GF Nan‐e‐Fasaee samples can be consumed by patients with CD, diabetes, high cholesterol, and obesity due to their low fat, carbohydrate, and calorie levels. Moreover, these GF samples can be a healthy functional bakery product because of their high nutritional value, especially in terms of ash, protein, and fiber content.

#### 3.4.2 Color evaluation

The surface color measurement of optimal samples has been done by the L*a*b* color scale, as illustrated in Table 4. Generally, color is considered as an important qualitative characteristic in determining the consumer acceptability of bakery products.

As illustrated in Table 4, the highest L*a*b* values in GF Nan‐e‐Fasaee samples were indicated in the control sample at 73.33 ± 4.27, 4.83 ± 0.4, and 27 ± 7.37, respectively. In other words, the control sample was brighter than the GF samples, which is related to the richer protein and ash content of QF (Cannas et al., 2020; Demir & Kılınç, 2017).

The light color of GF bakery products is usually considered an undesirable feature compared to ordinary bakery products (Cannas et al., 2020). So, it is suggested that the darker color of optimal GF Nan‐e‐Fasaee samples can be considered as one of the desirable features of this study.

Despite the predominance of Maillard reaction products in the color determination of bakery crust (Mohammadi et al., 2021; Shiri et al., 2021), similar trend has not been observed here, which is supposed to be induced by the dominance of quinoa color.

## 4 CONCLUSION

Optimization of quinoa‐based GF Nan‐e‐Fasaee formulations using different polymerization degrees of inulin seems feasible. Results indicated that the efficiency of inulin in providing structure for GF Nan‐e‐Fasaee is dependent on its content, polymerization degree, and the way they are incorporated; e.g., the impact of L‐inulin on hardness is found to be dependent on the sugar level. Overall, formulations containing S‐inulin up to 40% w/w as a sugar‐substituting agent and those containing S‐ and L‐inulin in combination at 30% and 10% w/w as sugar and fat replacers, respectively, are considered to be advisable to provide desirable technological, chemical, and nutritional characteristics of GF Nan‐Fasaee samples. The interference of S‐inulin in the structure formation of non‐gelatinized starch and the reduction of available water induced by L‐inulin incorporation are supposed to be the main reasons restricting the higher substitution of sugar and fat by S‐ and L‐inulin incorporation, respectively.

## AUTHOR CONTRIBUTIONS

**Narjes Jamali:** Formal analysis (equal); methodology (equal); writing – original draft (equal). **Mehran Sayadi:** Project administration (equal); supervision (lead). **Roghayeh Nejati:** Project administration (equal); supervision (lead). **Faezeh Mohammadi:** Formal analysis (equal); methodology (equal). **amene nematollahi:** Supervision (equal); writing – review and editing (equal). **Neda Mollakhalili Meybodi:** Supervision (equal); writing – review and editing (equal).

## FUNDING INFORMATION

No Funding has been received.

## CONFLICT OF INTEREST STATEMENT

The authors declare no conflict of interest.

## ETHICS STATEMENT

This study was approved by the Institutional Review Board of School of Public Health, Fasa University of Medical Sciences. Approval ID:IR. FUMS. REC. 1399.136.

## CONSENT FOR PUBLICATION

All authors agree to publish.

## Data Availability

The research data are not shared.
